# Redirecting NK cells to the lymph nodes to augment their lymphoma-targeting capacity

**DOI:** 10.1038/s41698-024-00595-w

**Published:** 2024-05-20

**Authors:** Laura Sanz-Ortega, Caroline Leijonhufvud, Lisanne Schoutens, Mélanie Lambert, Emily Levy, Agneta Andersson, Björn E. Wahlin, Mattias Carlsten

**Affiliations:** 1 Department of Medicine, Huddinge, Center for Hematology and Regenerative Medicine, Karolinska Institutet, Stockholm, Sweden; 2 Université Sorbonne Paris Nord, INSERM, Paris, France; 3 Cellular and Molecular Therapeutics Branch, National Heart, Lung, and Blood Institute, National Institutes of Health, Bethesda, MD USA; 4 Unit of Haematology, Department of Medicine, Huddinge, Karolinska Institutet, Stockholm, Sweden; 5 Center for Cell Therapy and Allogeneic Stem Cell Transplantation, Karolinska Comprehensive Cancer Center, Karolinska University Hospital, Stockholm, Sweden

**Keywords:** Immunotherapy, Genetic engineering, Lymphoma

## Abstract

CAR-NK cells can induce remission in lymphoma patients. We speculate that the full potential of adoptive NK cell immunotherapy against lymphoma is restricted by their poor lymph node (LN) homing capacity. Here, we have utilized a clinically approved transfection method with the aim of redirecting NK cells to LNs. Electroporation of ex vivo expanded NK cells with mRNAs coding for CCR7, CXCR5, and CD62L resulted in increased in vitro migration towards chemokines and mouse LN-derived supernatant. Following infusion into SCID/Beige mice, modified NK cells showed enhanced LN homing. Importantly, lymphoma patient-derived NK cells were equally well expanded and engineered as healthy donor NK cells, highlighting their translational potential. Additionally, the introduction of high-affinity CD16, together with the homing molecules, also augmented their ADCC capacity against autologous lymphoma cells. Hence, genetic engineering can be utilized to enhance NK cell LN homing. The homing concept may synergize with CAR- or monoclonal/bi-/tri-specific antibody-based approaches.

## Introduction

Natural killer (NK) cells hold promise for the treatment of cancer^1^. In recent years, early-phase clinical trials have confirmed their potential in settings of adoptive cell transfer. Although NK cell infusion has been explored in patients with malignancies such as multiple myeloma and ovarian carcinoma, the majority of clinical trials have so far been conducted in patients with myeloid leukemia, including acute myeloid leukemia (AML) and myelodysplastic syndromes (MDS)^2–4^.

Recently, Rezvani and colleagues showed that cord blood-derived NK cells engineered to express a CD19-directed chimeric antigen receptor (CAR) can induce remission in patients with advanced lymphoma^5^. In this pioneering study, the incorporation of *IL-15* within the introduced construct enabled long-term CAR-NK cell persistence via autocrine stimulation, overcoming their poor in vivo persistence. Nevertheless, NK cell homing to the lymphoma was not explored.

Strategies to improve NK cell-based immunotherapy have historically focused on augmenting cellular cytotoxicity, leaving trafficking of the effector cells to the tumor site unexplored^6^. However, in recent years, proper NK cell homing to the tumor has been identified as a potential limiting factor for efficient NK cell immunotherapy^7^. Efforts have been made to introduce chemokine receptors (CKRs) to NK cells and have shown promising preclinical results. Examples of this include the overexpression of CXCR2 on NK cells for improved migration towards renal cell carcinoma^8^ or the co-expression of CXCR1 and an NKG2D CAR to enhance the efficacy of NK cells against different solid cancers^9^. We have recently shown that AML and high-risk MDS patients who receive an adoptive transfer of NK cells with high surface expression of the bone marrow (BM) homing CKR CXCR4 have a higher probability of reaching a complete remission compared to patients who receive NK cells with lower expression levels of CXCR4^3^. Using animal models, we have also shown that the introduction of gain-of-function CXCR4 on NK cells can redirect these cells to the BM^10^. As lymphoma primarily develops in the lymph nodes (LNs), proper homing of adoptively infused NK cells to this anatomical site is likely central to mounting potent anti-tumor responses. Given that the lymphocyte population within the LNs of healthy individuals and lymphoma patients normally contains less than 1% NK cells^11,12^, there is room for increased infiltration.

One reason for the low number of NK cells in LNs is that only the small population of CD56^bright^ NK cells expresses the CKR CCR7, known to direct cells to secondary lymphoid tissues such as the LNs via its chemoattraction to CCL19 and CCL21^13^. The expression of the adhesion molecule CD62L (L-selectin), which is needed for cells to bind efficiently to specific ligands on high endothelial venules (HEVs) within LN tissues, is also confined to the CD56^bright^ NK cell subset^11,14^. Furthermore, NK cells do not normally express CXCR5, which is necessary for the chemoattraction to germinal centers via CXCL13^15^. Notably, the lack of CXCR5 expression may limit NK cell homing to the B follicles within the LNs^16^. However, interestingly, it was recently described that patients with lymphoma still had few NK cells (around 1%) in their lymphoma-involved LNs, but with a higher CD56^dim^/CD56^bright^ NK cell ratio^12^. In line with this, a mature CD56^dim^ NK cell population was predominantly found in tumor-infiltrated LNs in melanoma patients^17^, suggesting a potential role of this NK cell subtype within the LNs in cancer. This also indicates that there are likely additional mechanisms involved in controlling NK cell trafficking to and/or accumulation in the LNs.

As NK cells exert antibody-dependent cellular cytotoxicity (ADCC) via the CD16 receptor, they are therapeutic players in several currently used antibody-based treatment regimens for lymphoma. Cartron et al. and Weng et al.^18,19^ report that patients with follicular lymphoma (FL) homozygous for the high-affinity CD16 (158 V) allotype have an almost two-fold increased chance of responding to antibody therapy, and those responding have a twice as long duration of response. Other data such as the role for KIR-KIR ligand pairs on response duration following rituximab treatment of FL and increased NK cell numbers following IL-2 therapy have been shown to correlate with clinical responses of rituximab-treated non-Hodgkin’s B-cell lymphoma patients support a role for NK cell ADCC in the treatment of this disease^20,21^. However, direct contact of antibody-coated tumor cells with effector cells is crucial to achieving treatment responses. Hence, the limited expression of the proper CKRs, such as CCR7 and CXCR5, and key adhesion molecules, such as CD62L, may limit the contribution of NK cells in currently used treatment regimens for lymphoma as many of these are based on therapeutic antibodies, including the anti-CD20 antibody rituximab. Unfortunately, there are today no well-established xenograft lymphoma mouse models fully mimicking human lymphoma disease that reliably can be used to explore the role of NK cell homing to the LNs for tumor control.

Here we show that ex vivo expanded human NK cells engineered by mRNA electroporation to express the CKRs CCR7 and CXCR5 and the adhesion molecule CD62L have improved LN homing properties in vivo. The genetically engineered NK cells showed potent in vitro migration capacity without compromised viability and cytotoxicity, even when combining all three molecules. Importantly, NK cells from FL patients were equally efficiently ex vivo expanded and engineered with homing molecules as healthy donor NK cells. Their cytotoxicity against antibody-coated autologous lymphoma cells was further augmented by the addition of the high-affinity CD16 receptor. Overall, our data highlights homing as a new important modality that needs further attention in translational research and clinical trials on adoptive NK cell infusion to treat lymphoma. Future studies will have to explore the potential of redirecting NK cells to the lymphoma microenvironment.

## Results

### Efficient upregulation of key molecules for LN homing on expanded human NK cells following mRNA electroporation results in potent in vitro migration

**Fig. 1:**
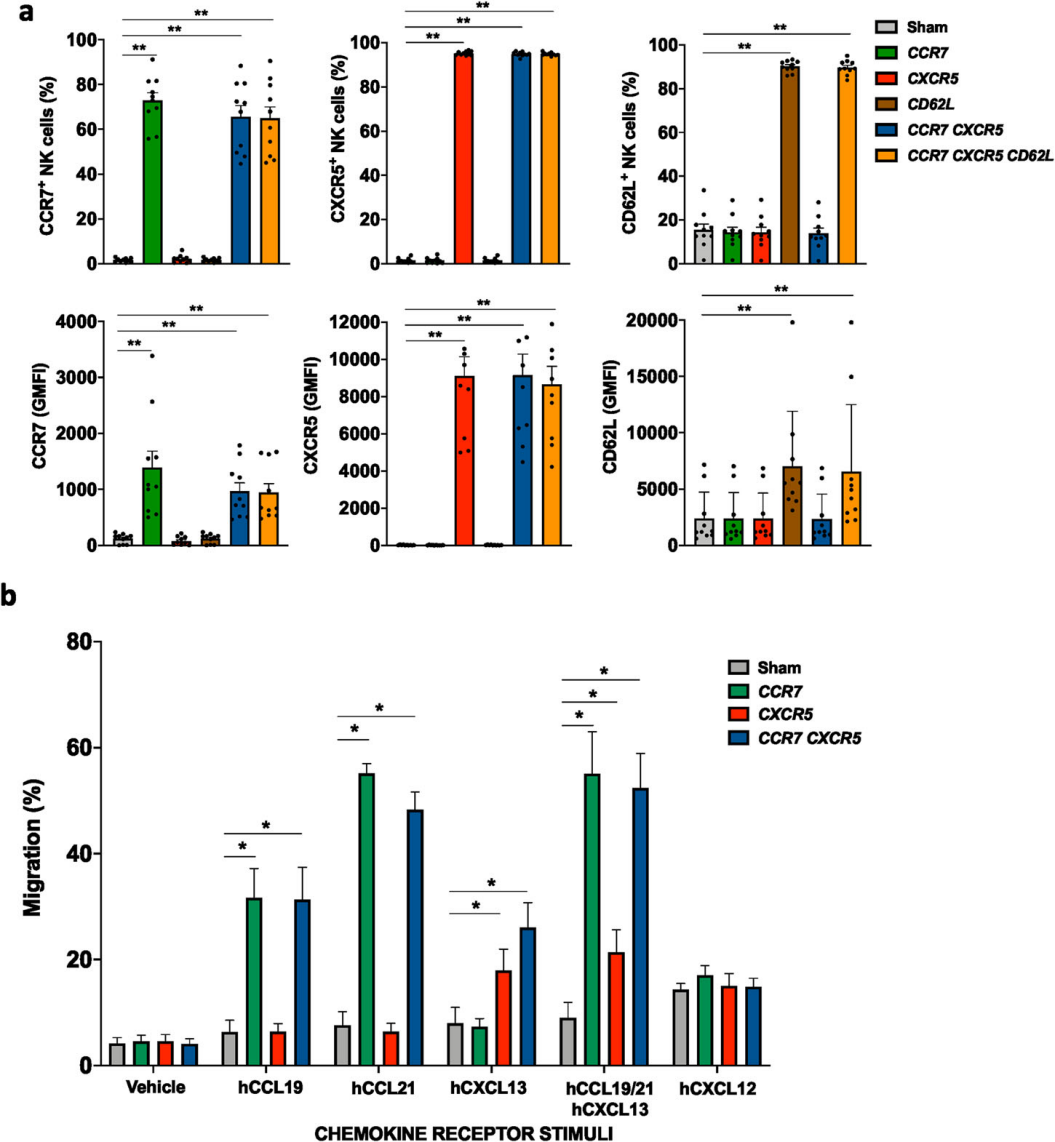
Efficient upregulation of key lymph node homing molecules on primary human NK cells following mRNA electroporation triggers prominent and specific migration. **a** CCR7, CXCR5, and CD62L expression levels (% and geometric mean fluorescence intensity (GMFI)) on ex vivo expanded human NK cells 6 hours after electroporation with one or several mRNAs (*n* = 10). **b** Transwell migration of Sham (no mRNA) or mRNA-electroporated human NK cells against gradients of one or several of the following chemokines: CCL19 (CCR7 ligand), CCL21 (CCR7 ligand), CXCL13 (CXCR5 ligand) and CXCL12 (CXCR4 ligand) (*n* = 6–10). Bars, mean. Error bars, SEM. The Wilcoxon matched‐pairs signed‐rank test comparing mRNA-electroporated vs sham-electroporated NK cells was used for statistics.

To explore whether the introduction of selected CKRs on human NK cells could help redirect their migration to LNs, we first characterize expression levels of endogenous molecules and the functional consequences following mRNA electroporation of the NK cells. To this end, we used ex vivo expanded NK cells that are highly cytotoxic against malignant cells and are currently being explored clinically (NCT00720785)^22^. As can be observed in Fig. 1a, the introduction of CCR7, CXCR5, or CD62L, and combinations thereof, resulted in high-frequency and high-intensity surface expression for the individual molecules, even when all three were introduced simultaneously. Molecules reached peak expression within less than 10 hours and were detectable for up to 24–48 hours following electroporation. Importantly, the introduction of one or several of these molecules did not impact the NK cell viability, CD56 expression, and the baseline expression of other central CKRs (Supplemental Fig. 1a–d). This adds to previous data showing that the proliferative capacity and expression of activating and inhibitory receptors by NK cells that have undergone mRNA electroporation remains largely unaltered^23^. Maintaining this phenotype is critical when designing a translational approach for redirecting NK cells to LNs. As highlighted in Fig. 1b, the introduction of CCR7 and CXCR5 led to potent in vitro migration towards corresponding chemokine ligands CCL19 and CCL21, or CXCL13, respectively. As expected by unchanged CXCR4 expression (Supplemental Fig. 1c), migration towards its ligand CXCL12 was unaffected by the transfection with *CCR7* and *CXCR5* mRNAs (Fig. 1b). Since CD62L is not involved in chemokine-mediated migration but instead mediates adhesion to the endothelium prior to migration, co-electroporation of mRNA coding for CD62L together with mRNA coding for CCR7 and CXCR5 did not affect the migration triggered by these CKRs in our in vitro assay (Supplemental Fig. 1e).

In summary, these data demonstrate that mRNA transfection of NK cells is an efficient method to transiently introduce one or several CKRs together with adhesion molecules on the NK cell surface without negatively affecting viability and phenotype while boosting their migration towards their corresponding ligands.

### The introduction of CCR7, CXCR5, and CD62L does not alter baseline NK cell cytotoxicity or ADCC

**Fig. 2:**
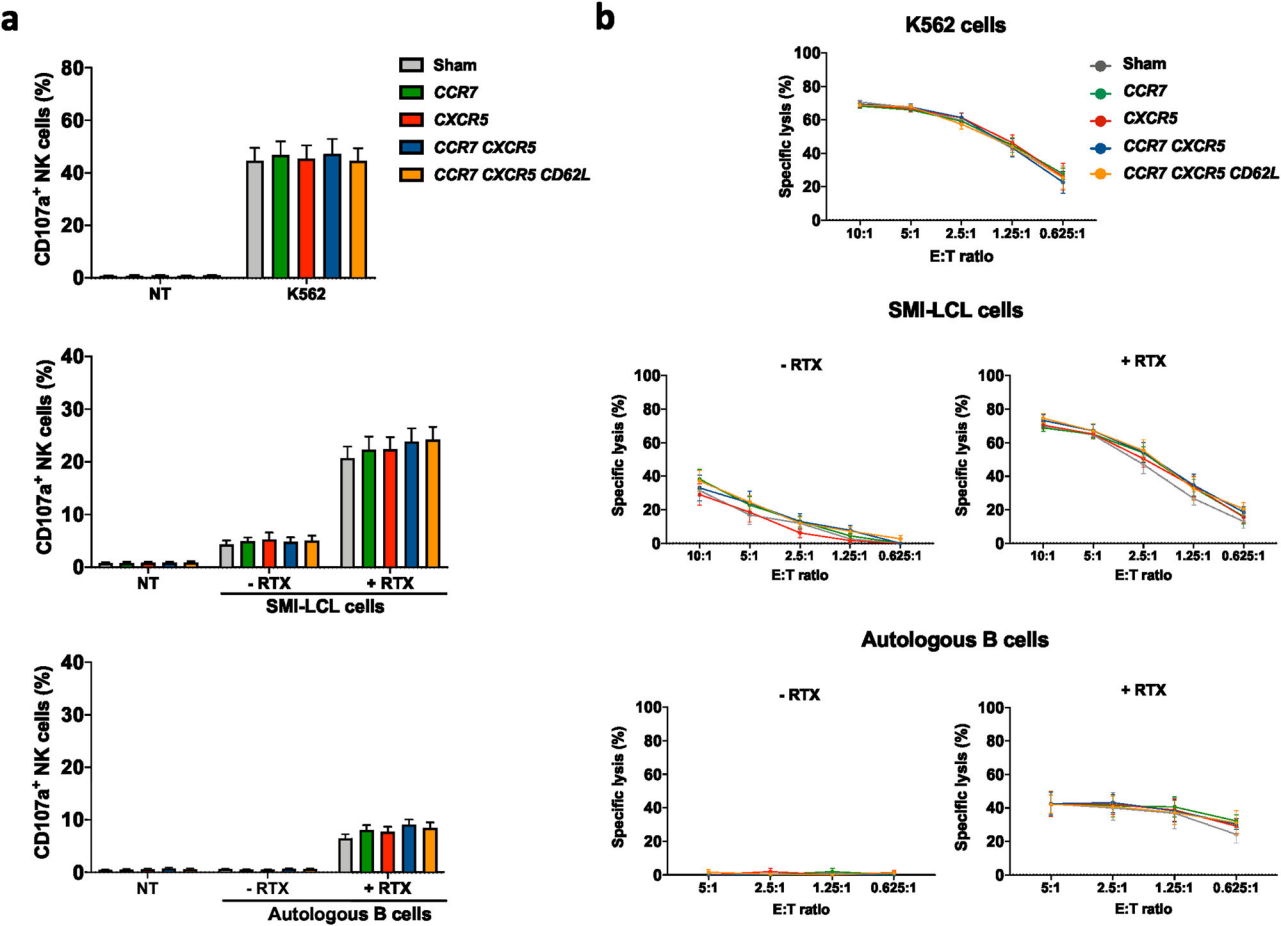
Unaltered baseline NK cell cytotoxicity and targeting via ADCC following the introduction of CCR7, CXCR5, and CD62L. **a** NK cell degranulation and **b** target cell killing following co-cultures with NK cells and the denoted target or without target (no target, NT) initiated 6 hours after electroporation of the NK cells with one or several mRNAs (*n* = 4–6). Co-cultures performed in the presence of rituximab are marked with +RTX. Bars/symbols, mean. Error bars, SEM. E:T ratio, Effector-to-Target ratio. The Wilcoxon matched‐pairs signed‐rank test was used for statistics.

Since the overall aim of this study was to direct intravenously infused NK cells to LNs to more efficiently target lymphoma cells in this anatomical location, we next addressed if degranulation and cytotoxicity of mRNA-modified NK cells were different from that of control NK cells. As shown in Fig. 2, the introduction of CCR7 or CXCR5 alone, or the introduction of both together with CD62L, did not impact the degranulation or general cytotoxic capacity of NK cells following co-culture with the gold standard NK cell target cell line K562 (Fig. 2). Since one of the major mechanisms of action for rituximab is ADCC^24^, we next co-cultured control and mRNA-electroporated NK cells with the EBV-LCL cell line SMI-LCL or isolated autologous B-cells in the absence or presence of rituximab. The degree of rituximab-triggered degranulation and elevated cytotoxic function was indifferent between mRNA-modified and sham NK cells when co-cultured with either SMI-LCL cells or autologous primary B-cells (Fig. 2). Accordingly, the CD16 expression levels remained unaltered after the mRNA electroporation with one or several mRNAs (Supplemental Fig. 2).

### Human CCR7 triggers the migration of NK cells towards mouse CCL19 and 21, whereas human CXCR5 poorly does towards mouse CXCL13

**Fig. 3:**
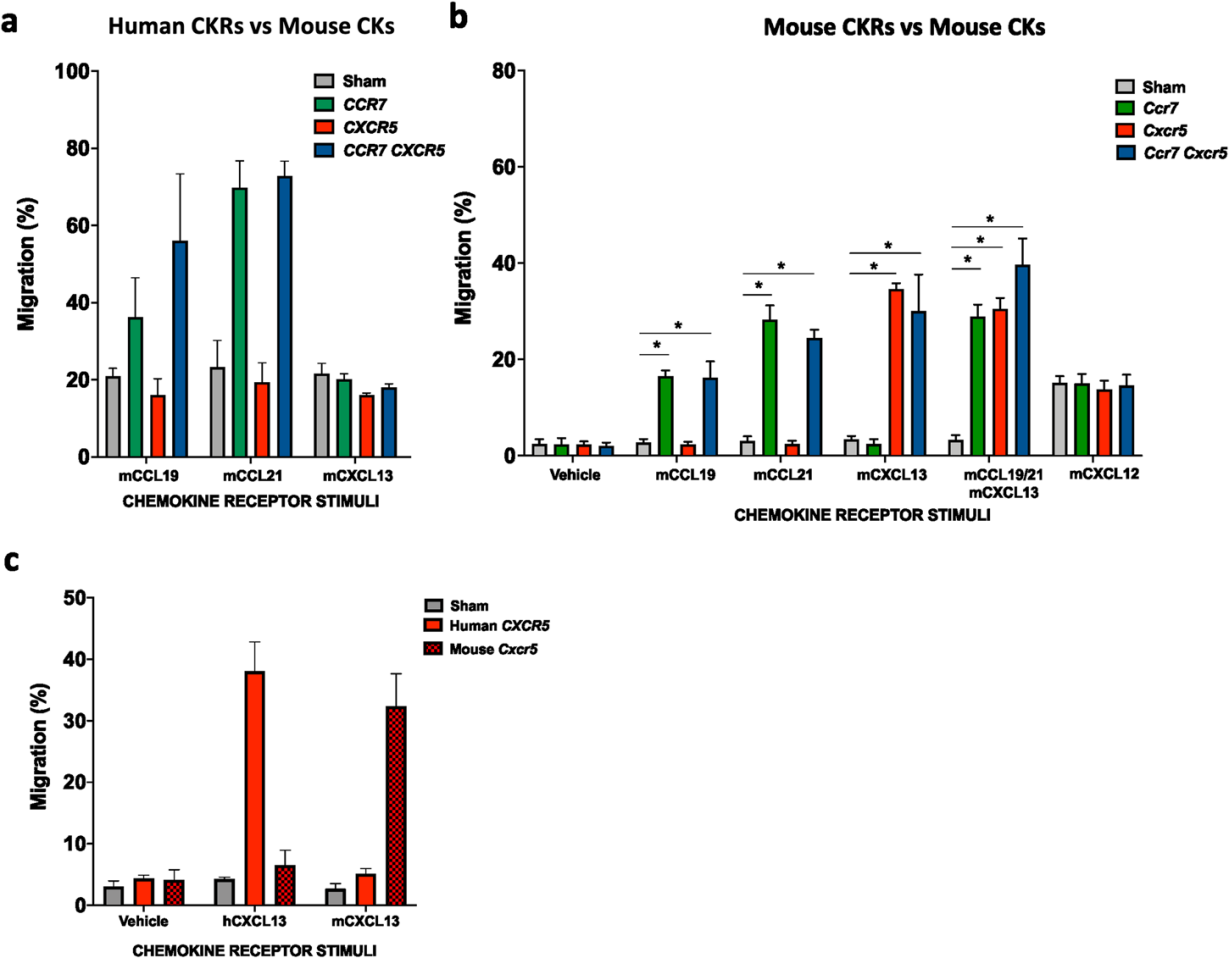
Human NK cells modified to express human CCR7 and CXCR5 migrates efficiently towards mouse CCL19 and CCL21 but not mouse CXCL13 due to poor homology between species for this chemokine. **a** Transwell migration of human NK cells against gradients of different mouse chemokines 6 hours after electroporation with mRNAs coding for human CCR7 and/or CXCR5 (*n* = 3) or **b** mouse CCR7 and/or CXCR5 (*n* = 6). **c** Differential migration of human NK cells towards human and mouse CXCL13 after electroporation with human *CXCR5* or mouse *Cxcr5* mRNA (*n* = 3). Bars, mean. Error bars, SEM. The Wilcoxon matched‐pairs signed‐rank test comparing mRNA-electroporated vs sham-electroporated NK cells was used for statistics.

Numerous CKRs and ligands are conserved between humans and mice^25^. However, protein alignment by the function Blast 2 sequences in blastp showed that CXCL13 is only conserved to 44% between the two species^26–28^. Therefore, before conducting in vivo experiments, we investigated the in vitro migration potential of expanded human NK cells electroporated with either human or mouse CKRs. Functional migration was assessed against either mouse or human chemokine ligand gradients (Figs. 1b and 3). From this, we observed that human NK cells overexpressing CCR7 alone or in combination with CXCR5 migrated well when exposed to CCL19 or CCL21; this held true for the human-human, mouse-mouse, and human-mouse axes. In contrast, human CXCR5 did not improve in vitro migration of NK cells towards mouse CXCL13, while both human and mouse CXCR5 did migrate towards the CXCL13 chemokine of congruent species (Fig. 3c). These data confirmed the lack of recognition for the human receptor towards the mouse ligand due to the substantial discrepancy between mouse and human CXCL13. This indicates that the optimal design for in vivo experiments requires the use of mouse CKRs.

**Fig. 4:**
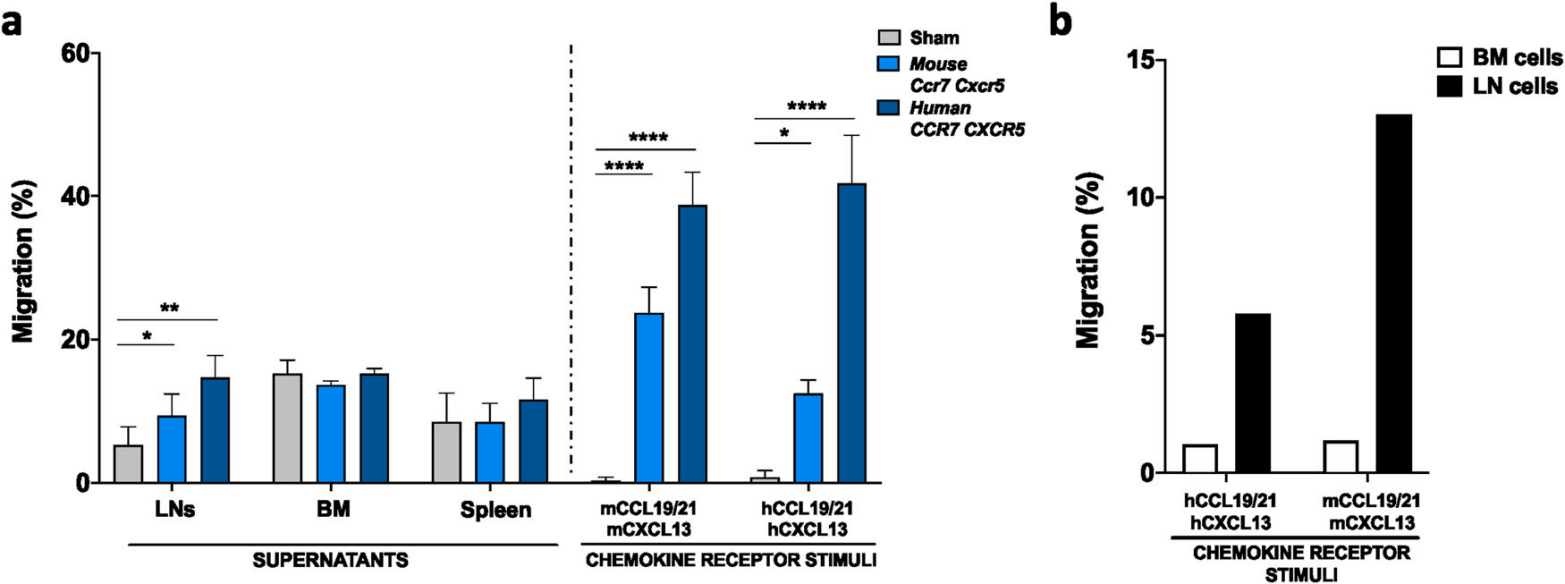
Primary human NK cells modified to express human or mouse CKRs have an increased migration potential towards supernatants of mouse LNs, whereas no change is observed for supernatants of other organs. **a** Experimental layout for ex vivo migration after electroporation. Ex vivo migration towards the supernatant derived from LNs, BM, and spleen obtained from Rj: ATHYM-Foxn1nu/nu (nude) mice as well as human and mouse chemokines 6 hours after electroporation of human NK cells with mRNAs coding for human or mouse LN homing molecules (*n* = 7–9). **b** Ex vivo migration of mouse cells derived from BM and LNs of nude mice towards the human or mouse chemokines. Bars, mean. Error bars, SEM. The Mann–Whitney *U* test comparing mRNA-electroporated vs sham-electroporated NK cells was used for statistics.

To further plan for the in vivo experiments, we electroporated expanded human NK cells with human *CCR7* and *CXCR5* or mouse *Ccr7* and *Cxcr5* mRNAs and conducted ex vivo migration assays against mouse BM, spleen, and LNs supernatants. As shown in Fig. 4a, there was a trend that expanded human NK cells expressing the two human CKRs migrated better to the mouse spleen and LN supernatant compared to NK cells expressing the corresponding mouse CKRs. However, according to our migration data towards the pure chemokine ligand control and expression kinetics of the respective CKRs on the NK cells (Fig. 4a and Supplemental Fig. 3), this difference was likely mainly related to the lower proportion and intensity of expression of the mouse receptors on the human cells compared to the human receptors (Supplemental Fig. 1b).

Next, to address the significance of mouse versus human receptors in migrating to LNs in mice, we isolated cells from the mouse BM and LNs and ran an in vitro migration assay toward either human or mouse CCL19/21 and CXCL13. This experiment revealed that the mouse LN cells were attracted to both human and mouse CCL19/21 and CXCL13 but had stronger chemoattraction to the mouse-derived molecules (Fig. 4b). In conclusion, these data indicate that the mouse CKRs are more appropriate for use in murine in vivo homing experiments. Based on this, we also decided to use the mouse CD62L molecule in future in vivo homing experiments to avoid potential species biases.

### Introduction of CCR7, CXCR5, and CD62L triggers in vivo LN homing of adoptively infused primary human NK cells

**Fig. 5:**
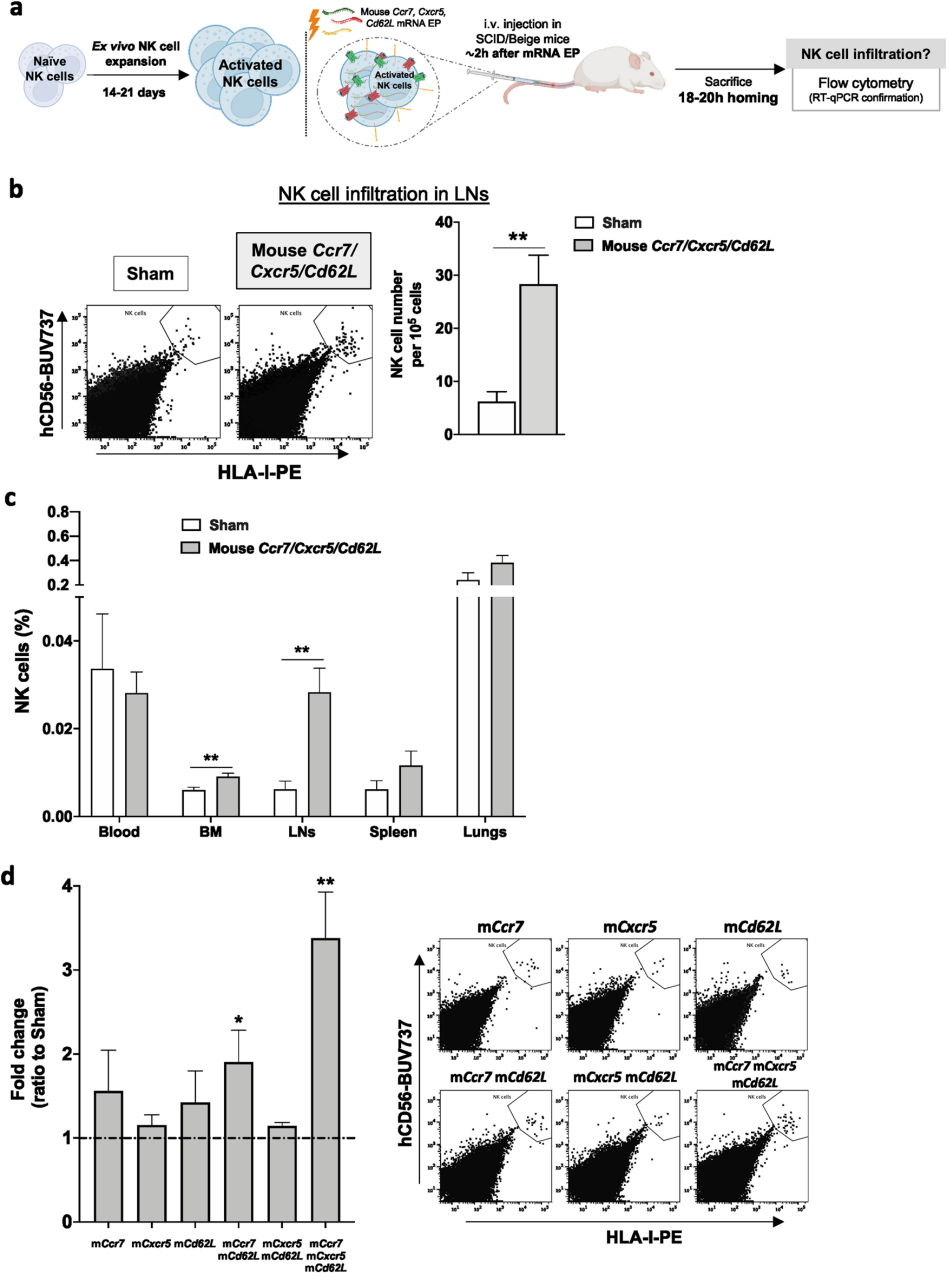
Upregulation of CCR7, CXCR5, and CD62L on human NK cells triggers increased homing to LNs in vivo. **a** Experimental layout for in vivo homing of adoptively infused expanded human NK cells electroporated with mRNAs coding for the mouse CCR7, CXCR5. and CD62L molecules. Created with BioRender.com. **b** Dot plots (pooled mice for each condition) for NK cell LN homing comparing Sham vs *Ccr7/Cxcr5/Cd62L* mRNA conditions and assessment of NK cell number in LNs by flow cytometry, calculated out of the total live cells (*n* = 9 mice). **c** In vivo homing of ex vivo expanded human NK cells in several organs 18–20 hours after cell transfer into SCID/Beige mice as assessed by flow cytometry, comparing Sham vs *Ccr7/Cxcr5/Cd62L* mRNA conditions (*n* = 9 mice). **d** In vivo homing of ex vivo expanded human NK cells in LNs after mRNA electroporation coding for one or several molecules compared to Sham (no mRNA) (*n* = 3–6 mice) and dot plots (pooled mice for each condition) for NK cell homing comparing all mRNA conditions. Bars, mean. Error bars, SEM. The Mann–Whitney *U* test comparing mRNA-electroporated vs sham-electroporated NK cells was used for statistics.

The overall goal of this study was to provide proof-of-concept that NK cells can be genetically engineered to have a higher propensity to migrate to LNs. To evaluate if our approach for NK cell modification could improve cellular homing into the LNs in vivo, we infused expanded human NK cells into SCID/Beige mice and assessed their infiltration by flow cytometry while the LN homing molecules were optimally expressed on the NK cell surface (Fig. 5, Supplemental Fig. 4a). Despite lacking mature T- and B-cells and functional NK cells, SCID/Beige mice have primitive LNs which contain the proper chemokine signature that make them suitable for this type of study^29,30^. Indeed, the presence of these chemokines together with other LN markers was assessed by RT-qPCR and flow cytometry to ensure the harvested tissue truly represented LNs (Supplemental Fig. 4b). 18–20 hours following intravenous infusion of non-mRNA and mRNA-electroporated expanded human NK cells, there was a significant increase of NK cells in the LNs of the mice receiving mouse CCR7, CXCR5, and CD62L-modified human NK cells compared to controls (Fig. 5b, c). The enhanced NK cell infiltration within the LNs was further confirmed by RT-qPCR (Supplemental Fig. 4c). As can be observed in Fig. 5c, there was a tendency for reduced numbers of circulating mRNA-modified NK cells and an increase of mRNA-modified NK cells in other tissues compared to control NK cells. Additionally, although the use of human receptors in mice could be more clinically relevant, and even if a similar migration reaction towards the LNs was observed when mRNAs coding for the human receptors were used, this was more prominent/robust when using the mouse variants of the receptors (Supplemental Fig. 4d). Hence, electroporation of mRNA coding for LN homing molecules can be utilized to direct human NK cells to the LNs of healthy mice.

To better understand the contribution of the individual molecules, we next performed experiments on modified NK cells expressing either a single homing molecule or a combination. Again, NK cells expressing all three molecules showed the most potent LN homing capacity when injected in vivo. As can be appreciated from Fig. 5e, both CCR7 and CD62L seemed to promote somewhat stronger LN homing compared to the other conditions involving either one or two molecules.

### FL patient-derived NK cells can be expanded ex vivo and modified to express LN homing molecules and high-affinity CD16 for improved targeting of autologous tumor cells

**Fig. 6:**
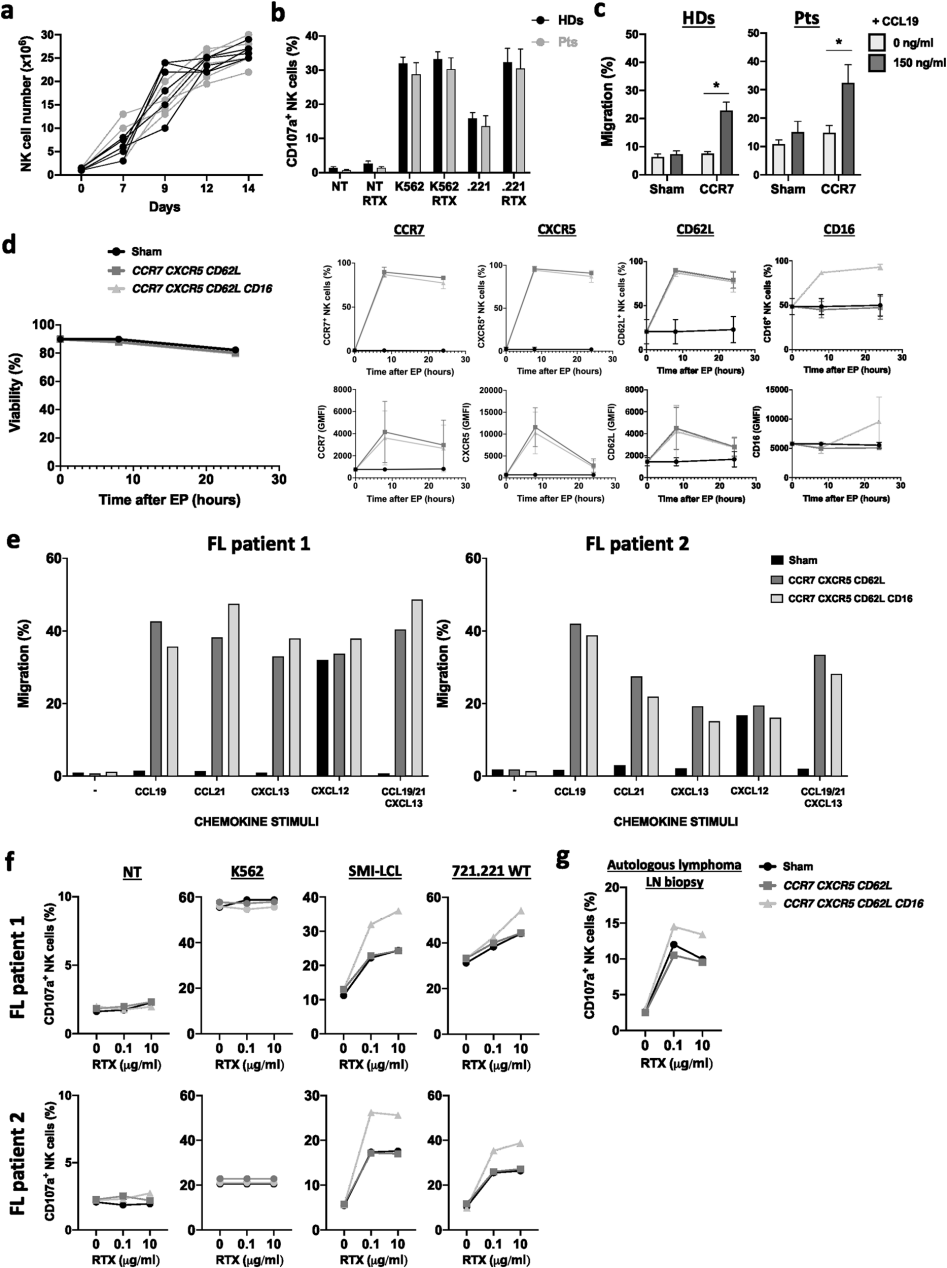
FL patient-derived NK cells can be successfully expanded and mRNA-electroporated for improved tumor targeting. **a** Ex vivo expansion and **b** degranulation capacity of NK cells from PB of patients with FL and healthy donors (*n* = 6). **c** Transwell migration of Sham (no mRNA) and *hCCR7* mRNA-modified NK cells against gradients of CCL19 6-8 hours after electroporation (*n* = 6). **d** Viability and expression kinetics of CCR7, CXCR5, CD62L, and CD16 molecules on ex vivo expanded human NK cells from FL patients after mRNA electroporation compared to Sham (no mRNA) (*n* = 2). **e** Transwell migration of Sham (no mRNA) and mRNA-electroporated NK cells from two FL patients against gradients of one or several chemokines 6–8 hours after electroporation. **f** Degranulation capacity of NK cells from two patients with FL against different target cell lines 24 hours after mRNA electroporation compared to Sham (no mRNA). **g** Representative example of degranulation capacity of ex vivo expanded human NK cells from a lymphoma patient against autologous LN fine-needle biopsy material containing primary tumor cells after electroporation with Sham, *CCR7, CXCR5, CD62L* or additionally *CD16-158V* mRNA. The Mann-Whitney U test was used for statistics in **a** and **b**.

To test if our proof-of-concept studies can be applied to a clinical setting, we expanded and modified PB NK cells from patients with FL. Indolent FL patients are often treated with anti-CD20 monotherapy, making this tumor type interesting as a translational model to further explore. Considering that NK cells are important effector cells in antibody therapy, this group of patients could potentially benefit from the co-administration of engineered NK cells and a monoclonal antibody, a BiKE or a TriKE, or by administering CAR-NK cells equipped with LN homing molecules. As shown in Fig. 6a, the expansion potential of NK cells from PB of patients with FL was equal to that of PB NK cells from healthy donors. Patient NK cells had equal cytotoxicity towards K562 cells and the CD20^+^ EBV-LCL cell line 721.221, with and without rituximab (Fig. 6b).

Next, we addressed whether expanded patient NK cells can be manipulated to express a CKR for enhancing migration towards the corresponding ligand in vitro. We focused on CCR7 as this receptor triggered the most potent LN migration in the previous experiments. This experiment revealed that patient-derived ex vivo expanded PB NK cells, similar to healthy donor-derived NK cells, could be engineered with CCR7 to trigger equally potent in vitro migration to CCL19 (Fig. 6c). Overall, this data demonstrates that PB NK cells from patients with FL can be ex vivo expanded and manipulated to direct migration, similar to healthy donor-derived PB NK cells. These findings suggest the strong translational potential of our approach.

Lastly, we aimed to show that patient NK cells could be manipulated to gain improved activity against autologous lymphoma cells via ADCC at the same time as increased LN homing potential. To this end, we transfected expanded NK cells from two patients with *CCR7, CXCR5, CD62L,* and high-affinity *CD16* mRNAs. Electroporation with three or four different mRNAs did not impact the viability of the NK cells. Introducing high-affinity CD16 increased surface CD16 expression and did not affect the expression kinetics of the LN homing molecules (Fig. 6d, Supplemental Fig. 5). Similar to healthy donor-derived NK cells, the introduction of both CCR7 and CXCR5 in the patient NK cells led to higher in vitro migration towards the corresponding chemokine ligands CCL19, CCL21, and CXCL13 and did not impact their response to CXCL12 (Fig. 6e). This migration was not affected by introducing other molecules such as CD62L and high-affinity CD16. NK cells engineered to express the high-affinity CD16 receptor along with the LN homing molecules showed more degranulation when co-cultured with SMI-LCL or 721.221 WT, but not K562, in the presence of 0.1 or 10 μg/ml rituximab, confirming specifically enhanced ADCC (Fig. 6f). Importantly, this boost in ADCC by high-affinity CD16 overexpressing NK cells was also observed when co-cultured with autologous lymphoma cells acquired from fine-needle biopsy material of one of the patient (Fig. 6g).

## Discussion

NK cell cancer therapies have recently gained significant attention for their ability to induce remission in certain patients^1^. Positive clinical outcome following CAR-NK cell therapy was recently published, highlighting how NK cells can be armed with a CAR for improved tumor targeting and with autocrine IL-15 production for improved in vivo persistence^5^. Although these NK cells get the accurate signals to persist and efficiently target the tumor, proper homing of the adoptively infused NK cells to the tumor site represents a challenge that has yet to be more comprehensively addressed^31^. This paper shows that NK cells can efficiently be manipulated to express key homing molecules, resulting in improved trafficking to LNs when infused in vivo, where lymphoma and metastases of certain solid tumors often reside. Moreover, our paper reports that PB NK cells from lymphoma patients can be expanded and modified equally efficiently as healthy donor-derived NK cells. We also believe that redirecting NK cells to the tumor site has the potential to augment current approaches that aim to improve anti-tumor cytotoxicity. Based on our findings, we propose that homing should be considered in future strategies to enhance the efficacy of adoptive NK cell immunotherapy.

There is a large amount of evidence highlighting the association between the grade of NK cell tumor infiltration and the clinical outcome of specific tumor types^32^. However, the therapeutic potential of actively redirecting adoptively infused NK cells to the tumor site has not been fully explored. In a clinical trial to assess the adoptive transfer of short-term IL-2 activated NK cells in treating patients with AML and high-risk MDS performed at Karolinska University Hospital, patients who received NK cells with higher endogenous expression of the BM homing receptor CXCR4 had a higher likelihood of responding compared to those receiving a product with lower cell surface levels of endogenous CXCR4^3^. This indirect evidence, claiming a positive correlation between NK cell BM homing potential and clinical response in myeloid leukemia, has been validated in retrospective analyses of myeloid leukemia patients undergoing adoptive NK cell infusion where a higher degree of NK cell infiltration in the BM correlated to better clinical response^32^. The more direct evidence of the role of redirected tumor homing of adoptively infused NK cells comes from our recent preclinical in vivo data showing that NK cells manipulated to overexpress the gain-of-function variant of CXCR4, CXCR4-R334X, have enhanced AML clearance in the BM of mice compared to AML-bearing mice receiving control NK cells^33^. This and other similar data^9,34^ underline the role of proper infiltration of adoptively infused NK cells to attack tumors better and indicate that proper homing of the NK cells is critical to enable efficient CAR- and antibody-mediated enhancement of the anti-tumor cytotoxicity by the NK cells.

In the present study, we did not aim to address the anti-tumor potential of redirected NK cell homing to the LNs. Instead, the focus was to generate a method and provide proof-of-concept for utilizing genetic engineering to destine a significantly higher proportion of the adoptively infused NK cells to the LNs in a tumor-free situation. In this tumor-free scenario, we observed that CCR7 and CD62L seemed to promote stronger LN homing compared to the other conditions explored involving only one or two of the selected molecules in this study. This confirms that CD62L and CCR7 are key in the entry to the LN while CXCR5 is not. However, the most significant increase in LN infiltration was found when all three molecules, CCR7, CXCR5, and CD62L, were introduced. We hypothesized that these triple mRNA-electroporated NK cells use CD62L and CCR7 to infiltrate within the LN, and once inside, both CCR7 and CXCR5 interact with their corresponding ligands, which promotes higher retention in the LN compared to the mRNA-electroporated NK cells lacking CXCR5. Despite lacking follicular pattern organization, LNs from SCID/Beige mice present the complete chemokine signature^30^, which could allow these interactions.

As discussed in the paper, the homing signature of a lymphoma-involved LN is likely different from that of a healthy LN. CCR7 and CD62L, as well as their corresponding ligands, are known to have an essential function in distinct blood cancers and the metastatic spread of solid tumor cells through the LNs^35–38^. At the same time, CD62L-mediated NK cell recruitment has been shown to control tumor metastasis within secondary lymphoid organs^39^. Upregulation of CXCR5 and its ligand CXCL13 is also common in B-cell lymphomas^40,41^. This proves the importance of the molecules used in this study and supports their exploitation even under the lymphoma context. Moreover, it has been reported that non-lymphoid tumor tissues can also present HEVs similar to those found in LNs^42^. These tumor associated-HEVs are, in line with LN HEVs, known to be associated with lymphocyte infiltration. Hence, the approach shown here may therefore also be explored to enhance lymphocyte trafficking to solid tumors or even to other non-lymphoid organs where lymphoma has spread. However, other molecules can further contribute to the homing of the tumor microenvironment of lymphomas. The latter are the CCR4 ligands, CCL17 and CCL22, which are upregulated in FL^43^, T-cell non-Hodgkin’s lymphomas^44^, and classical Hodgkin’s lymphoma^45^, whereas the CXCR4 ligand, CXCL12, is found in several non-Hodgkin’s lymphomas^46–48^. Besides this, CXCL9 and CXCL10, which are CXCR3 ligands, have been shown relevant in T-cell lymphomas^44^ and classical Hodgkin lymphoma^45^. Therefore, future studies can aim to elucidate if there is a universal CKR expression signature that can be used for redirecting infused cells to lymphoma-involved LNs, or if analyses of biopsies or extirpated whole LNs are required for a personalized approach. Regardless, our study provides proof of concept that one can easily combine mRNA coding for several CKRs with mRNA coding for adhesion molecules to better target the cells to the anatomical site once infused in vivo without affecting viability, phenotype, and function.

Our study has limitations. Ideally, one would explore the potential of the redirected homing in a lymphoma model to establish proof-of-concept for increased targeting of lymphoma in the LNs and provide evidence for improved overall survival in the same model. However, the field currently lacks proper lymphoma mouse models with tumor growth in the LN compartments, which is a prerequisite for fully addressing this approach. Xenografted mice with human lymphoma growth unnaturally in non-LN organs after subcutaneous or intravenous inoculation are currently being used^49^. Some studies have used models in which the lymphoma cell line is manipulated to overexpress LN-associated chemokines such as CCL19 or CXCL13 to address the anti-tumor potential of CCR7-engineered CAR NK92 cells^50^ or CXCR5-engineered CAR-T cells^51^, respectively. These models, however, do not recapitulate tumor growth within the LNs and, therefore, cannot be used to assess immune cell infiltration in lymphoma-bearing LNs. Hence, neither of these models would be adequate to address the in vivo tumor targeting capacity of our NK cell product programmed for redirected homing to the LNs. Consequently, we conclude that our approach must be explored in patients to generate a final proof-of-concept along with insights into any potential side effects. Another possible limitation when studying the impact of this concept is the transient nature of homing molecule expression following mRNA electroporation. On the other hand, transient expression provides a potentially safer product that could be used in clinical translation to assess the safety of modified NK cells infused into patients. Today, given that very little is known in the field, it is unclear if stable expression of CCR7 and/or CXCR5 along with CD62L is needed or if a transient expression is enough to drive the cells to the LNs and then other mechanisms help them sequester. These dynamics are yet to be explored. A potential advantage with the mRNA approach is that it is efficient and in contrast to, for instance, the trogocytosis approach used by Somanchi et al. ^52^. It is already now clinically applicable if using the MaxCyte instrument. It can also efficiently be used as ‘plug’n play’, where a given combination of molecules dictated by the location of the tumor or the chemokine signature can be introduced without adverse effects^23^.

Although our study provides proof-of-concept for genetically engineering NK cells to home to the LN, additional molecular pathways can likely be modified to further enhance the trafficking to the LNs. The introduction of different homing molecules can likely refine the homing to the LN per se and potentially even to the precise anatomical site within the LN^53^. Another potential aspect of our efforts that may need to be further explored in the future is the competing attraction by other homing molecules, such as other CKRs and/or adhesion molecules. On this end, we found S1PR5 to be lowly expressed on our NK cell product, hence not promoting strong attraction back to circulation^54,55^. However, the extravasation process involves the tethering/rolling, chemoattraction, firm adhesion, and transmigration phases. Most likely, as discussed before, additional targets in this process can be explored along with what is presented in this study to further improve the redirection to the lymphoma or solid tumor metastasis-involved LNs. For example, previous studies employing the same ex vivo expansion protocol as used in this study showed an increased potential for cells to migrate to solid melanoma tumors following upregulation of CXCR3^56^, which could be appreciated in our data as well. Therefore, CXCR3 knockout for disrupting this migration axis may improve homing to other tissues, in cases where the ligands are irrelevant in the lymphoma environment. Similar approaches have been explored for CCR5 on NK cells^57^. Further work is needed to fully understand how this best can be utilized in the setting of adoptive cell cancer therapy.

The significance of actively redirected homing of adoptively infused NK cells for cancer treatment has emerged from preclinical animal models. However, this potential in the clinical context is yet to be established. Furthermore, the synergistic effect of directed homing and CAR or high-affinity CD16 expression with monoclonal antibody therapy is unknown. Based on data from Ng et al.^34^, where CXCR4 redirected CD38 CAR-expressing NK cells presented an enhanced BM homing in myeloma-carrying mice or where the co-expression of CXCR1 and the NKG2D CAR on NK cells was used to improve their efficacy against solid tumors^9^, one could expect this to at least be additive. However, future studies are required to dissect when and how to utilize this for the most optimal anti-tumor effect.

In summary, these data establish proof-of-concept that adoptively infused NK cells can be redirected to the LNs using the combination of CCR7, CXCR5, and CD62L. To the best of our knowledge, this is the first study revealing the individual and combination effect of these molecules in directing NK cells to the LNs following infusion. Given the advancement of NK cell cancer immunotherapy and the recent development in lymphoma, we believe homing is an understudied modality that can help improve the overall approach, especially for lymphoma with a poor presence of NK cells in LNs. As signals most likely differ from those identified under normal physiological homeostasis, future studies need to elucidate what receptor-ligand interactions govern the redirection of the NK cells to the lymphoma microenvironment. Our paper provides a platform for a ‘plug’ n play’ pipeline for the exploration of the role of various CKRs and adhesion molecules, which will guide future translational efforts to define the clinical impact of this approach.

## Methods

### Healthy donors and patient samples

Peripheral blood (PB) cells were collected from healthy blood donor buffy coats following written informed consent (Ethical approval Dnr 2006/229-31/3). Indolent lymphoma patients were recruited following written informed consent (Ethical approval Dnr 2012/783-31/3), after which PB and fine needle aspirations from LNs were collected. PB mononuclear cells (PBMCs) were isolated by high-density gradient centrifugation before being cryopreserved. The use of human material and data in this work complied with all relevant ethical regulations, including the Declaration of Helsinki.

### Isolation and expansion of NK cells

NK cells were isolated from thawed PBMCs using the human NK cell isolation kit (Miltenyi) for magnetic bead separation according to the manufacturer’s protocol. Ex vivo expansions were performed according to previously published protocol^22^.

### Cell lines and reagents

All cell lines were cultured in complete media of RPMI 1640 (Gibco) supplemented with 10% FBS (Gibco). The K562 cell line was purchased from ATCC. The SMI-LCL cells were a kind gift from Dr. Childs, NIH, USA. The 721.221 WT cell line was a kind gift from Dr. Parham, Stanford, USA.

### Flow cytometry

Cells were labeled with the following anti-human antibodies and reagents for flow cytometry. Anti-CD3 (1:25; UCHT1 (561416)), anti-CD16 (1:100; 3G8 (563692)), anti-CD56 (1:50; NCAM16.2 (345811)), anti-CD19 (1:50; SJ25C1 (349449)), anti-CCR7 (1:50; 150503 (562555)), anti-CD62L (1:400; DREG56 (741843)), anti-CXCR1 (1:25; 5A12 (743421)), anti-CXCR4 (1:200; 12G5 (563924)), anti-CX_3_CR1 (1:100; 2A9-1 (565796)), anti-CCR5 (1:50; 2D7 (612808)) and BD^TM^ CompBeads were purchased from BD Biosciences. Anti-CXCR5 (1:25; 51505 (FAB190A)) and anti-S1PR5 (1:25; 282503 (FAB3964N)) were purchased from R&D Systems. Anti-CXCR3 (1:50; G025H7 (353732)), anti-CCR1 (1:200; SF10B29 (362913)), anti-CD107a (1:50; H4A3 (328644)), anti-HLA class I (1:50; W6/32 (311406)), and the Zombie NIR™ Fixable Viability Kit and the TruStain FcX™ PLUS (anti-mouse CD16/32; 1:100 (156603)) antibody, were all purchased from Biolegend. Fixable dead cell marker LIVE/DEAD Aqua was purchased from Invitrogen eBiosciences. When corresponding, the following anti-mouse antibodies were used to label the cells. Anti-CD45.2 (1:50; 104 (562895)), anti-CCR7 (1:400; 4B12 (562675)), and anti-CXCR5 (1:100; 2G8 (560615)) were purchased from BD Biosciences, and anti-CD62L (1:25; MEL4 (104412)) from Biolegend. Flow cytometry stainings were performed at 4 °C for 15–20 minutes, except for CKR staining, which was performed at 37 °C for 25–30 minutes. The BD Symphony A5 Special Order Research Product (BD Biosciences) was used to acquire the samples, and data were analyzed using the FlowJo software (BD Biosciences).

### NK cell transfection

2–4 μg/10^6^ cells of mRNA were transfected into ex vivo expanded NK cells using the MaxCyte GT electroporation instrument. Custom-made mRNAs encoding for human CCR7 and CXCR5 were obtained from TriLink Biotechnologies. mRNAs encoding for human CD62L and mouse CCR7, CXCR5, and CD62L were prepared from plasmids purchased from SinoBiological: (human CD62L (HG11838-UT), mouse CCR7 (MG50873-UT), mouse CXCR5 (MG50843-UT), mouse CD62L (MG50045-UT)) using the HiScribe T7 ARCA mRNA Kit (with tailing) and the MEGAclear™ Kit (Invitrogen) according to the manufacturer’s instructions.

### Degranulation assay

mRNA or sham (no mRNA) electroporated NK cells and target cells were co-cultured at an effector-to-target (E:T) ratio of 1:1 for 1 hour at 37 °C. NK cells without target cells were used as negative control detecting spontaneous background activity. Following incubation, cells were stained with fluorescently conjugated antibodies for CD107a expression and markers to identify NK cells. ADCC was measured in co-cultures containing either 0.1 or 10 μg/mL of rituximab (Roche). Autologous healthy B-cells were isolated on the same day of the experiment using the human B-cell isolation kit (Miltenyi). Autologous patient lymphoma cells from fine-needle aspirations were also thawed on the same day as the assay but were not subject to further cell isolation due to limitations in cell number.

### Killing assays

mRNA or sham (no mRNA) electroporated NK cells were co-cultured with target cells with and without 10 μg/mL of rituximab (Roche) for 4 hours. Tumor killing was assessed using a Calcein-AM-based assay as previously described^58^.

### In vitro migration assay

Migration assays of human NK cells were performed 6–8 hours post mRNA transfection. One or a combination of recombinant human or mouse chemokines (CCL19 (100 ng/mL), CCL21 (100 ng/mL), CXCL12 (100 ng/mL), and CXCL13 (200 ng/mL), all from Biolegend) were resuspended in 10% FBS-supplemented PBS (Gibco) and added to the bottom of plate wells. 1 × 10^5^ NK cells suspended in PBS were added into Corning Transwell® inserts placed into the wells with chemokines. Cells were incubated and allowed to migrate through the insert membrane for 1–2 h at 37 °C, after which the inserts were carefully removed. Migrated cells in the bottom were collected and quantified using the CyQuant kit according to the manufacturer’s instructions (Invitrogen). Fluorescence intensity was measured on a microplate spectrometer (Infinite® 200 PRO, Tecan). The proportion of migrated NK cells was calculated based on maximum control of NK cells placed directly at the bottom of the wells.

For ex vivo migration assays, BM, LNs, and spleens were harvested from Rj:ATHYM-Foxn1^nu/nu^ (nude) mice (Janvier Labs). The organs from 3 mice were pooled to prepare the supernatants in each experiment. The tissues were dissociated, and the cells and supernatants were collected separately. The supernatants were then filtered through a 0.4 μm pore-size filter (Millipore) before being used in the migration assay.

### Cellular homing assays in vivo

Animal experiments were performed under ethical approval (ID1533) by Jordbruksverket, Sweden. Two hours after mRNA electroporation, 10×10^6^ NK cells were injected intravenously into six to eight-week-old CB17.Cg-*Prkdc*^*scid*^*Lyst*^*bg-J*^/Crl (Fox Chase SCID Beige) mice, purchased from Charles River. Animals received 2 × 10^5^ IU of IL-2 intraperitoneally immediately after injection of the NK cells. Mice were sacrificed by cervical dislocation, and BM, LNs, spleen, and blood were harvested from animals 18–20 hours post injection. Tissues were dissociated and analyzed by flow cytometry. Human NK cells were identified, within the live cell population, by expression of human CD56, HLA class I, and mouse CD45 (live^+^ hCD56^+^ HLA-I^+^mCD45^−^), and NK cell number or percentage was calculated out of the total live cells in each tissue. In some experiments, RNA was isolated from cells harvested from the LNs and BM and pooled from several mice within the same group using the RNeasy Micro Kit (Qiagen). The High-Capacity cDNA Reverse Transcription Kit and the SYBR Green Master Mix, both from Applied Biosystems, were used to perform RT-qPCR using non-specific and human GAPDH primers as previously described^59^. Primers coding for the LN markers are as follow: *Cxcl13*, 5′-CATAGATCGGATTCAAGTTACGCC-3′ (forward) and 5′-GTAACCATTTGGCACGAGGATTC-3′ (reverse); *Ccl19*, 5′-TCGTGAAAGCCTTCCGCTACCT-3′ (forward) and 5′-CAGTCTTCGGATGATGCGATCC-3′ (reverse); *Ccl21*, 5′-CAAGGGCTGCAAGAGAACTG-3′ (forward) and 5′-TGTGAGTTGGACCGTGAACC-3′ (reverse); *Glycam1*, 5′-AAGACTCAGCCCACAGATGCCA-3′ (forward) and 5′-CTCTGAAGATGGAAGGCTCCTTG-3′ (reverse); and *Chst-4*, 5′-CGGATGTGTTCTACCTGATGGAG-3′ (forward) and 5′-CACAGGAAGACGGAACGCAGAA-3′ (reverse).

### Statistical analysis

Statistical analyses were performed using the GraphPad Software. The Wilcoxon signed-rank test was used to assess significance in paired non-parametric datasets, and the Mann–Whitney *U* test was used for unpaired non-parametric datasets. Significant results were marked by *p < 0.05, **p < 0.01, ***p < 0.001, and ****p < 0.0001 while non-significant results were not further specified.

### Reporting summary

Further information on research design is available in the Nature Research Reporting Summary linked to this article.

### Supplementary information

REPORTING SUMMARY

Supplemental Figures 1–5

## Data Availability

All data generated or analyzed during this study are included in this published article and its supplementary information files. It is available from the corresponding author under request.
