# Supplementary material for: Redirecting NK cells to the lymph nodes to augment their lymphoma-targeting capacity
Source: NPJ Precis Oncol. 2024 May 20;8:108. doi: 10.1038/s41698-024-00595-w (PMC11106342; doi:10.1038/s41698-024-00595-w)
Supplement: Supplementary file 2 — Supplemental Figures 1–5 [file 41698_2024_595_MOESM2_ESM.pdf]

Supplemental Figures and Figure Legends for

Redirecting NK-cells to the lymph nodes to augment their lymphoma-targeting capacity

Laura Sanz-Ortega[1], Caroline Leijonhufvud[1*], Lisanne Schoutens[1*], Mélanie Lambert[1,2], Emily Levy[3], Agneta Andersson[1], Björn E Wahlin[4] and Mattias Carlsten[1, 5#]

[1]Department of Medicine, Huddinge, Center for Hematology and Regenerative Medicine, Karolinska Institutet, Stockholm, Sweden. [2]Université Sorbonne Paris Nord, INSERM, France. [3]Cellular and Molecular Therapeutics Branch, National Heart, Lung, and Blood Institute, National Institutes of Health, Bethesda, MD, USA. [4]Unit of Haematology, Department of Medicine, Huddinge, Karolinska Institutet, Stockholm, Sweden, [5]Center for Cell Therapy and Allogeneic Stem Cell Transplantation, Karolinska Comprehensive Cancer Center, Karolinska University Hospital, Stockholm, Sweden.

*Equal contribution

Supplemental Figure 1

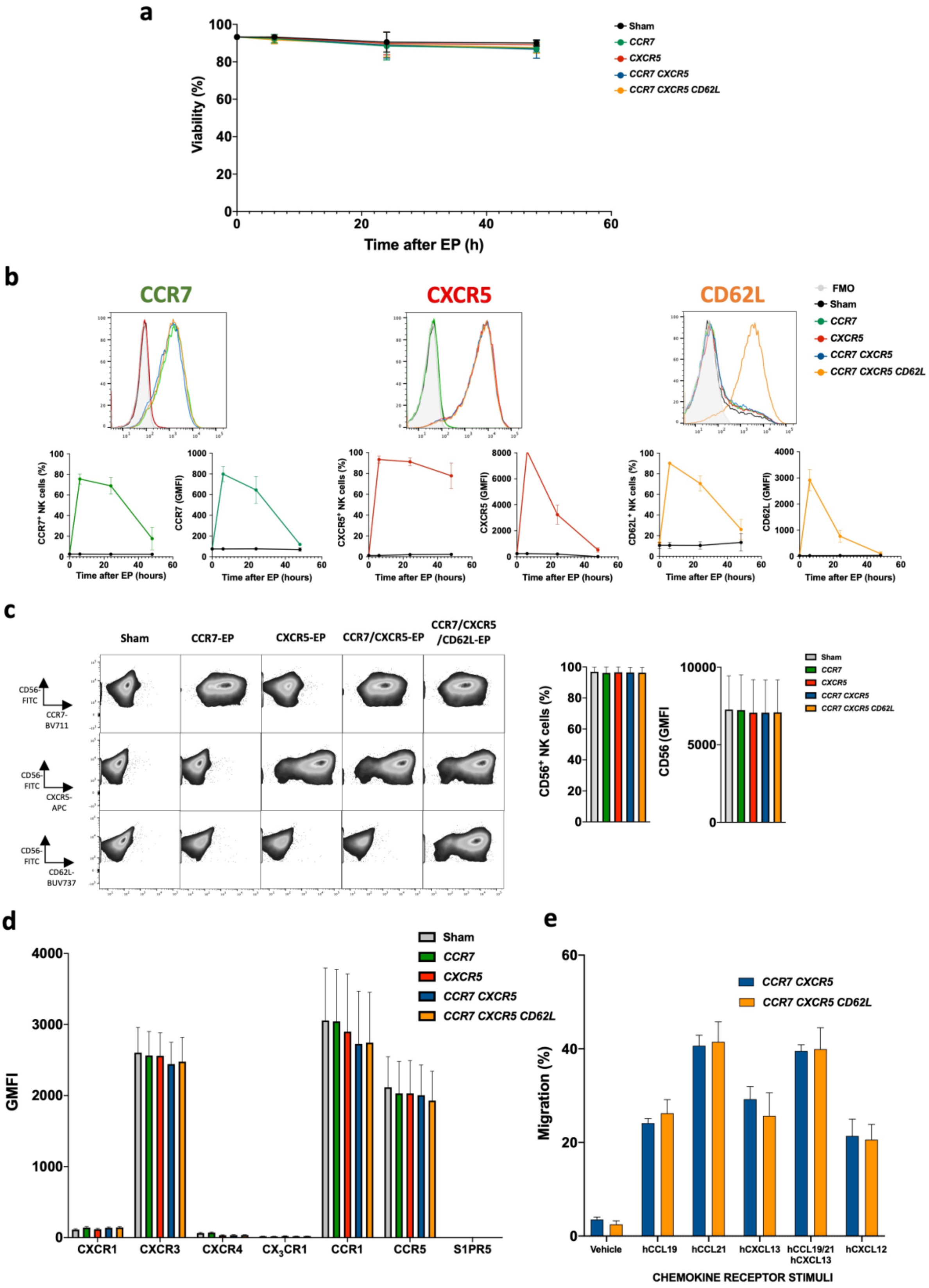

Supplemental Figure 1. Viability, expression kinetics for each molecule, phenotypic characterization and migration potential of *ex vivo* expanded human NK cells following mRNA electroporation. a) Viability of *ex vivo* expanded human NK cells from healthy donors following electroporation with 2-4 µg of one or several mRNAs per million NK cells. Sham (no mRNA) (n = 3). b) Representative histograms showing CCR7, CXCR5 and CD62L expression levels 6 hours after electroporation and graphs showing the expression kinetics (%) on *ex vivo* expanded human NK cells for up to 48 hours post electroporation (n = 3). c) Representative dot plots illustrating CCR7, CXCR5 and CD62L expression levels in the context of CD56 expression 6 hours after electroporation and graphs showing the corresponding CD56 expression levels (NK cells (%) and GMFI) on *ex vivo* expanded human NK cells in the different conditions (n = 3). d) Expression intensity (GMFI) of relevant chemokine receptors on human NK cells as assessed 6 hours after electroporation with the corresponding mRNA. (n = 3). e) Transwell migration assessed 6 hours showing that CD62L introduction does not interfere with migration of CCR7/CXCR5-mRNA electroporated NK cells (n = 6). Bars/symbols, mean. Error bars, SEM.

Supplemental Figure 2

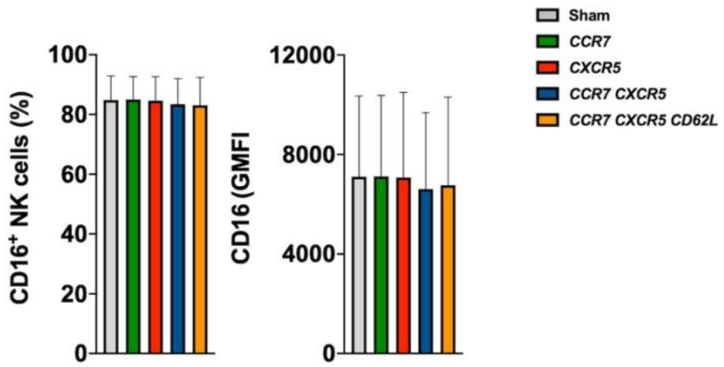

Supplemental Figure 2. CD16 expression levels on *ex vivo* expanded human NK cells following mRNA electroporation. CD16 expression levels (% and GMFI) on *ex vivo* expanded human NK cells 6 hours after electroporation with one or several mRNAs (n = 3). Symbols, mean. Error bars, SEM.

Supplemental Figure 3

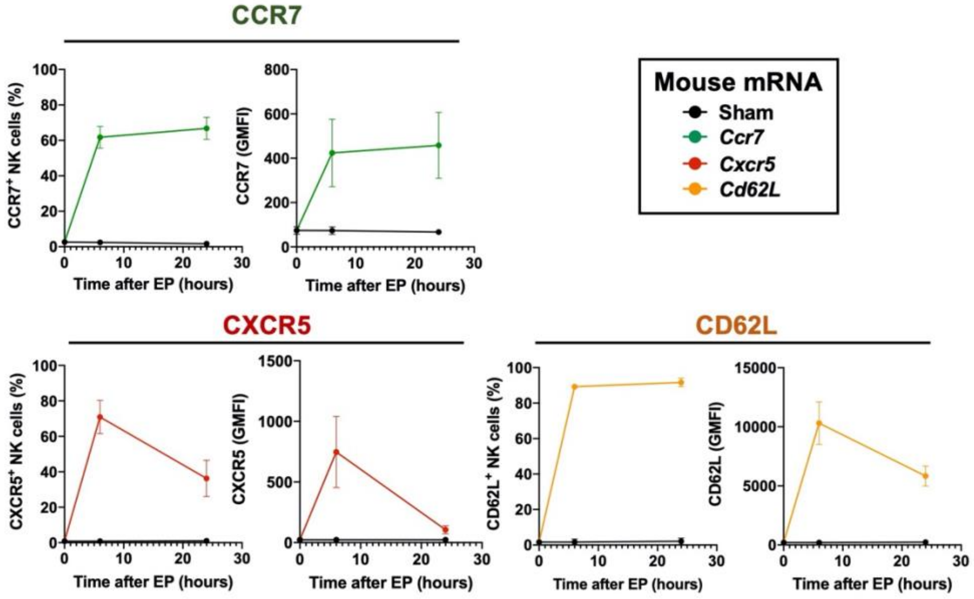

Supplemental Figure 3. Expression levels and kinetics of mouse CCR7, CXCR5 and CD62L on human NK cells. Expression kinetics of mouse CCR7, CXCR5 and CD62L molecules on human NK cells electroporated with the corresponding mRNAs compared to Sham (no mRNA) electroporated NK cells (n = 3-4). Symbols, mean. Error bars, SEM.

Supplemental Figure 4

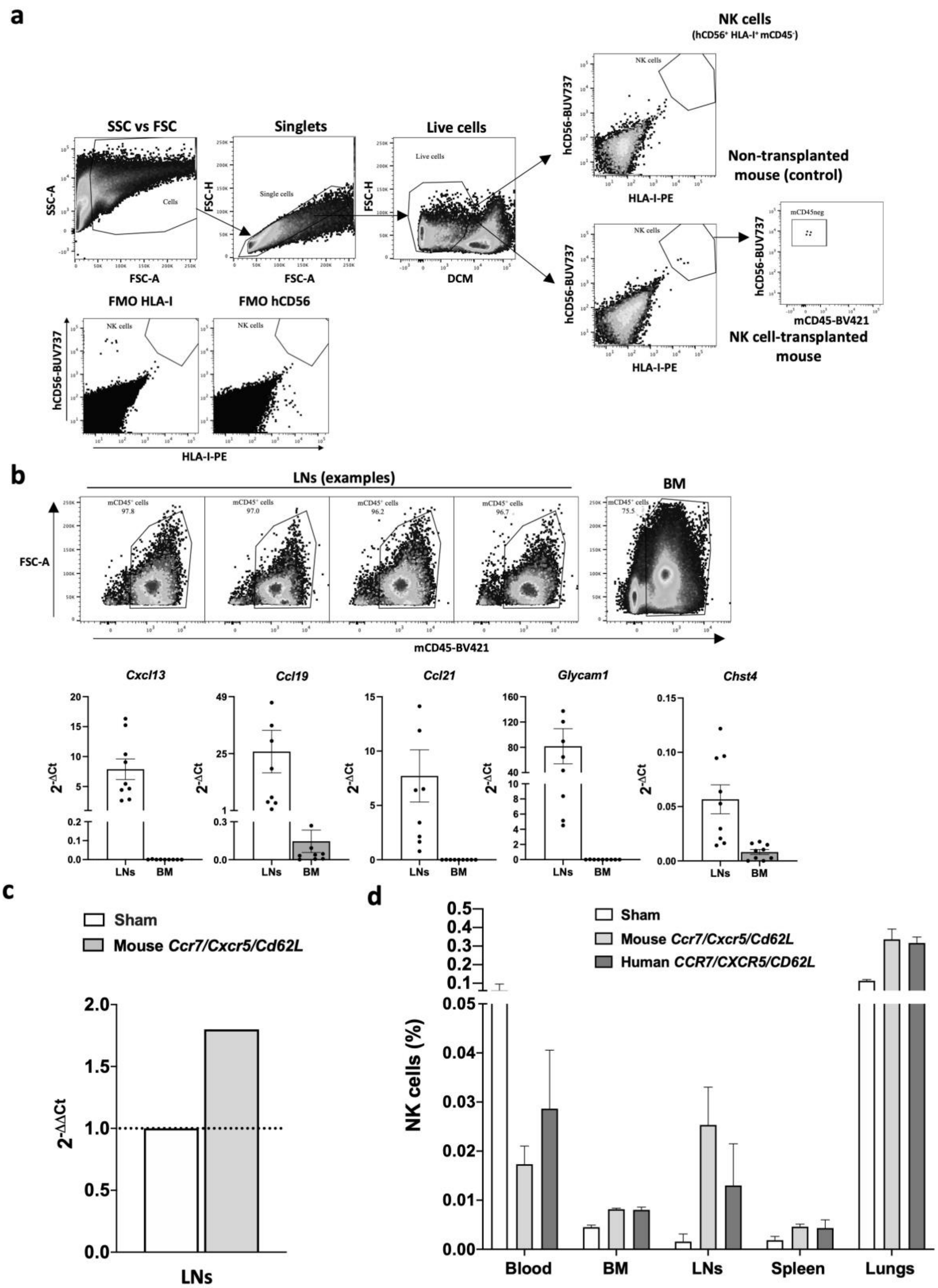

Supplemental Figure 4. Assessment of mRNA-modified NK cell infiltration in the LNs by FACS and verification by RT-qPCR. a) Gating strategy for human NK cells (live$^+$ hCD56$^+$ HLA-I$^+$mCD45$^-$) within mouse LNs and representative dot plots from one non-transplanted mouse (negative control) and one NK cell-transplanted mouse. Fluorescence minus one (FMO) staining controls are shown for the NK cell detection antibodies. b) Representative dot plots showing mouse CD45 staining in the harvested LNs compared to BM and relative quantification of LN markers in LNs and BM, using RT-qPCR of mRNA expression levels of *Cxcl13,Ccl19,Ccl21, Glycam1* and *Chst4*, normalized with the expression of the reference gene (mouse *Gapdh*) (n = 4-6 mice). c) Relative quantification of Ccr7/Cxcr5/Cd62L-modified human NK cells in mouse LNs using RT-qPCR of mRNA expression levels of human-specific GAPDH, normalized with the expression of the reference gene (non-specific mouse *Gapdh*) and compared with non-modified human NK cell infiltration. d) *In vivo* homing of *ex vivo* expanded human NK cells in several organs 18-20 hours after cell transfer into SCID/Beige mice as assessed by flow cytometry, comparing Sham, mouse *Ccr7/Cxcr5/Cd62L* and human *CCR7/CXCR5/CD62L* mRNA conditions (n = 3 mice).

Supplemental Figure 5

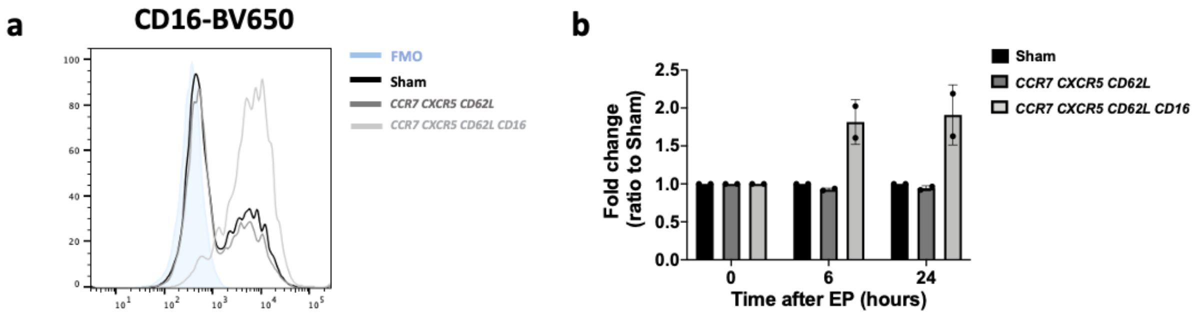

Supplemental Figure 5. CD16 expression at baseline and after mRNA electroporation. a) Representative histograms of CD16 expression 24 hours after mRNA electroporation. b) CD16 intensity of expression on CD16$^+$ NK cells from lymphoma patients at different time points after mRNA electroporation compared to Sham (no mRNA) (n = 2).
